# Humanitarian-specific recommendations for gender-transformative parenting programming: lessons from the field to address gender-based violence

**DOI:** 10.1016/j.eclinm.2024.102954

**Published:** 2024-11-27

**Authors:** Melissa Meinhart, Ilana Seff, Kathryn Falb, Julianne Deitch, Danielle Roth, Catherine Poulton, Lindsay Stark

**Affiliations:** a Washington University in St. Louis, 1 Brookings Dr, St. Louis, MO 63130, USA; b Johns Hopkins Bloomberg School of Public Health, 615 N Wolfe St, Baltimore, MD 21205, USA; c Women’s Refugee Commission, 15 West 37th Street, 9th Floor, New York, NY 10018, USA; d International Rescue Committee, 263 W 38th St, New York, NY 10018, USA; e UNICEF, United Nations Plaza, New York, NY 10017, USA

**Keywords:** Gender-based violence, Humanitarian settings, Adolescent girls, Parenting, Programming

## Abstract

There has been tremendous progress in building and promoting evidence-based practice around parenting programming in low- and middle-income countries. However, there remains a dearth of evidence specifically examining gender transformative programming designed to address gender-based violence in humanitarian settings. To inform this gap, we examine how existing gender transformative programmatic material addresses the unique circumstances of parenting in humanitarian settings. Incorporating lessons from the field, we inform considerations of how to adapt future content to address gender-based violence in humanitarian settings. We reviewed two gender transformative programs in humanitarian settings: Safe at Home and Sibling Support for Adolescent Girls in Emergencies. Four thematic recommendations emerged for gender-transformative parenting programming in humanitarian settings to address gender-based violence, specifically intimate partner violence and violence against children. These recommendations include: 1) Recognize the diversity of families in humanitarian settings, 2) Prioritize participatory approaches from the start, 3) Set realistic parameters and goals for the specific humanitarian context, and 4) Ensure pathways to scale and sustainability within the initial program design. We advocate for broader application of these principals to support gender-transformative parenting programming that is tailored to address gender-based violence in humanitarian settings and that will continue to build the respective evidence base.

**Search strategy and selection criteria**

The original authorship team, from Washington University and UNICEF, began with the goal of conducting a scoping review to identify gender-transformative programming that addressed gender-based violence in humanitarian settings. The team conducted a formal database search (See Fig. 1) within four databases, using five benchmark articles to support confirmation of search saturation. Reviewed programs were identified to be gender-aware and/or gender-transformative, including their respective components. Data were identified by searches of Pubmed, SocIndex, PsychInfo, Scopus, and references from relevant articles using the search terms “Parent∗ OR “Family functioning” OR “Whole-family” OR Caretak∗ OR “Child development” OR Caregiv∗” AND “Intervention OR program∗ OR guidelines” AND “Displac∗ OR Migrant OR “Natural disaster” OR Humanitarian OR Refugee”. Contact was made with program officers or authors of all eligible programming. Only materials published until 2023 were included. After reviewing 1699 resources, however, only two programs emerged from the extensive search: Safe at Home and the Sibling Support for Adolescent Girls in Emergencies (SSAGE) intervention (See Fig. 2). Recognizing that the field is too nascent for a full scoping review, we thus invited authorship from both programs to draw from our shared learning in this work to contribute as a Viewpoint. Collectively, we propose four key recommendations for humanitarian practitioners to consider when designing programs, supported by applied examples for each recommendation (Detailed in Table 1). The recommendations presented in this Viewpoint were developed with programs in mind that are feasible to be implemented within the constraints of humanitarian settings in order to reduce GBV, specifically IPV and VAC, while also centering participant and community voices for long-term sustainability (Table 2).

**Fig. 1:**
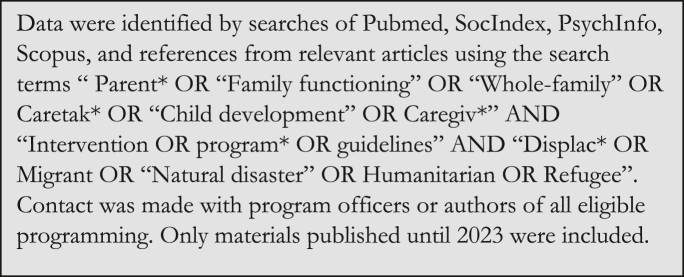
**Selection panel**.

**Fig. 2:**
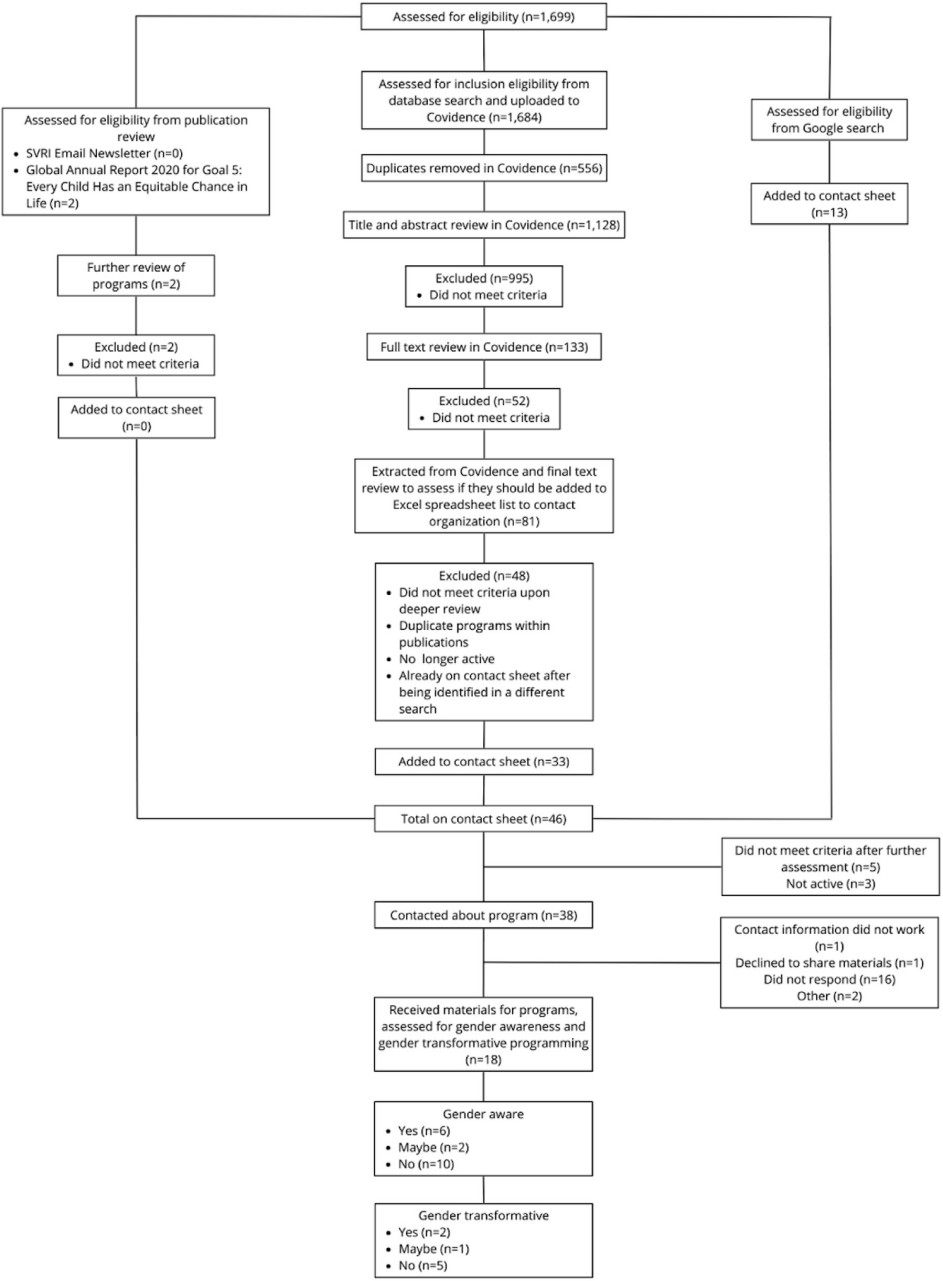
**Consort diagram**. Note: “not active” means that there was no active contact information for the respective program.

## Introduction

Forced displacement - a migratory movement with many drivers that minimally involves force, compulsion, or coercion^1^ - continues to surpass levels previously documented in history, contributing to a growing attention to responsive and effective programming for impacted populations. An emerging body of evidence demonstrates the effectiveness of parenting and caregiver interventions in improving parenting behaviors, mental health, and child well-being for forcibly displaced populations.^2^ Yet, there remains a paucity of evidence examining how parenting programming can address gender-based violence (GBV) in humanitarian settings, where the confluence of conflict, displacement, and various forms of vulnerability can magnify GBV risks and prevalence.^3^^,^^4^ While some have argued that GBV prevention should not be a priority due to the potential inadvertent risks to women in the absence of strong protection systems and referral pathways, there are also opportunities that arise from changes to the social order that tend to occur during disasters. Recognizing that these contextual shifts also provide an opportunity to impact behavior change and social norms,^3^^,^^5^ gender-transformative parenting programs (GTPP) may be especially impactful in humanitarian contexts, particularly to address intimate partner violence (IPV) and violence against children (VAC), which frequently co-occur. Shared drivers of household violence, including IPV and VAC, in humanitarian settings include: exposure to conflict, use of alcohol and drugs, strained social support and income, and adverse mental health.^6^ Gender-transformative parenting programs can serve to catalyze helpful shifts by explicitly examining gender norms, stereotypes, and the power dynamics that shape family and societal behaviors. While ‘gender-aware’ programming acknowledges differences in outcomes and access across genders, ‘gender-transformative’ approaches actively seek to change the underlying policies, programs, and structures that contribute to these differences.^7^ According to UNICEF, gender-transformative programming “… applies key principles such as gender equality and inclusion [and promotes] positive gender norms and socialization, in order to transform imbalanced power structures in families”.^8^ As such, gender-transformative parenting programming can empower individuals, especially caregivers, to challenge and transform the deeply ingrained gender norms that may perpetuate violence both directly and indirectly. Gender-transformative parenting programming can foster healthier and safer family dynamics, particularly in the context of intimate partner violence (IPV) and violence against children (VAC).

Previous efforts to synthesize learning from parenting programs in low and middle-income country contexts offer a starting point for understanding the unique potential of GTPP, including guidelines from the World Health Organization (WHO) on parenting interventions to prevent maltreatment and enhance parent-child relationships.^9^ These guidelines stress that evidence-based parenting interventions in humanitarian contexts should be readily accessible to all parents and caregivers. At the same time, WHO acknowledges that the evidence base in humanitarian settings is relatively weak and points to a crucial gap in comprehending how parenting programs might address IPV across settings. Meta-analysis of 14 parenting studies in humanitarian settings, however, found a small effect on physical and psychological violence,^10^ indicating the potential for parenting programming to address violence. A complementary evidence review from the Prevention Collaborative (2019) provides an examination of the efficacy of parenting programming in addressing violence within households, along with subsequent briefs.^11^, ^12^, ^13^ One of the five considerations for curriculum content includes promoting gender-equitable relationships in the family. This recommendation aligns with the evidence pointing to the potential of gender-transformative approaches, particularly when engaging fathers (UNICEF, 2023), a strategy with potentially promising implications for preventing GBV. In the pursuit of enabling gender-transformative parenting programming in humanitarian settings, UNICEF’s Resource Package for gender-transformative parenting also emerges as a valuable resource. While this package is not exclusive to humanitarian settings nor expansive in its considerations of GBV, it puts forth four gender-transformative parenting modules that could be refined for humanitarian contexts. These modules encompass one general module and three more tailored to different age groups.^14^ While the resource package identifies the importance of contextually adapting the modules, specific considerations for in humanitarian emergencies are not detailed. This adaptability is vital, as humanitarian contexts present unique challenges and complexities that necessitate tailored approaches. These resources offer a blueprint for implementing GTPP, yet further guidance is needed for implementation in humanitarian settings, in recognition that these contexts present unique challenges and complexities that necessitate tailored approaches.

In an effort to advance progress toward effective GTPP to address IPV and VAC in humanitarian settings, we reviewed materials and evidence from two gender transformative programs in humanitarian settings: Safe at Home and the Sibling Support for Adolescent Girls in Emergencies (SSAGE) intervention (See Table 1). We invited authorship from both programs to draw from our shared learning and engagement in this work. Collectively, we propose four key recommendations for practitioners to consider when designing such programs, supported by applied examples for each recommendation (see Table 2). The recommendations presented in this viewpoint were developed with programs in mind that are feasible to be implemented within the constraints of humanitarian settings in order to reduce GBV while centering participant and community voices for long-term sustainability.

**Table 1:** Overview of Safe at Home and SSAGE programming.

	Safe at home	Sibling support for adolescent girls in emergencies (SSAGE)
Overview and aims	Prevent and respond to IPV and child maltreatment in conflict-affected communities	Prevent mental illness, distress, and experiences of gender-based violence among adolescent girls in humanitarian settings
Target population	Heterosexual, monogamous couples and with at least one child aged 6–12 years	Adolescent girls and three of their family members: adolescent brother, male caregiver, female caregiver
Program content and design	Asset-based community-based discussion group series including weekly sex-segregated discussions with men and women (n = 24 sessions for men; n = 18 session for women) with five family sessions with children involved. Sessions take place across 6 months. Drawn from feminist and child development theories.	Gender-transformative, 12-session program utilizing a “whole family approach.” An adolescent girl, her male sibling, and a male and female caregiver participate in sessions that are age- and gender-specific and combined with family-wide discussions of session learnings. The sessions are engaging and prompt self-reflection and discussion on topics such as power, gender, interpersonal communication, and healthy relationships.
Contexts of implementation	Protracted humanitarian settings where there is stability of population movement for at least 6 months during the program implementation period. To date, it has been implemented in conflict-affected communities in the Democratic Republic of Congo, Myanmar, and in refugee camps in Tanzania.	With forcibly displaced populations. To date, it has been implemented in Northeast Nigeria with internally displaced peoples, with refugees in Niger, and with Syrian refugees in Jordan.

**Table 2:** Recommendations in practice.

Recognize the diversity of families in humanitarian settings (SSAGE & Safe at Home)	• In its first three cycles of implementation (Nigeria, Niger and Jordan), SSAGE required the adolescent male participant to be an older sibling of the participating adolescent girl. During the planning stages for an upcoming implementation of SSAGE with Venezuelan migrants in Colombia, implementing partner staff shared that the nature of displacement meant that adolescent girls often lived with other adolescent male relatives, such as cousins. As such, it was decided that inclusion criteria for adolescent males would be flexibly expanded to account for these realities and ensure inclusion of diverse household compositions. • In Niger, it was uncommon for households to have a married male and female caregiver. SSAGE program staff felt it would be exclusionary to only invite participants with both a male and female caregiver and thus only used the criteria of a male and female sibling. Program content was adjusted to focus on different types of caregiver-child relationships. • Based on demand from families, additional modules were developed as supplements to the core Safe at Home programming, including addressing the needs of people with disabilities. Additionally, there is specialized content for girl children. Tools are included within Safe at Home such as the power ladder which examines how different family members hold different positions within the family.
Prioritize participatory approaches from the start (SSAGE)	• In Niger, participatory contextualization processes for SSAGE revealed the need to create an image toolbox to accompany the curricula, as literacy of both program facilitators and participants was limited. Because SSAGE was the first intervention related to gender norms and roles in these communities, terms related to gender equality had to be introduced more slowly and with very specific contextual examples. • In Jordan, the majority of program participants had already been exposed to similar kinds of programming in the nearly ten years since being displaced from Syria. As such, program implementers worked to present SSAGE content in creative ways to avoid repeating information that participants were already familiar with. In some cases, the Jordan curriculum drew heavily from a popular Syrian TV program to discuss issues related to masculinity and harmful gender norms. • In Nigeria, participatory contextualization revealed how nuanced certain terms related to GBV were, which was further challenging due to the multi-lingual environment. Program facilitators received additional training on how to explain certain terms in the two different languages spoken in the communities. • In DRC, deep qualitative research, previous pilot tests, and engagement with practitioners continually refined the program before it was delivered as part of a randomized controlled trial evaluation.
Set realistic parameters and goals for the specific humanitarian context (Safe at Home & SSAGE)	• For Safe at Home, two major events disrupted program implementation. First, the initial months of the COVID-19 pandemic saw all ‘non-essential’, immediately life-saving services grind to a halt. Programming was disrupted for three months and when participants were able to meet again the numbers of people who could be together in a space was greatly reduced. Nevertheless, the demand for the program was high in communities, so Safe at Home groups were cut in half and program participants were scheduled for morning or afternoon sessions according to their availability. This required program implementers to be nimble in their approach, but the increased time burden on facilitators should be appropriately analyzed and compensated for. Second, the Nyiragongo volcano erupted in May of 2021, directly impacting the program sites and displacing the program participants. This caused a pause in the endline data collection and subsequent knock-on effect on the available timeline for implementation with the control group. Safe at Home program implementers decided to hold focus groups with participants who had already been through the program to gain their perspectives on which sessions in the curriculum were most impactful and essential. The Safe at Home team then re-designed the approach with a shortened curriculum which was delivered to the participants in the control group. • Balancing the imperative of prioritizing girls’ safety against the need to promote their mobility and freedom is a constant tension, recognizing that overemphasizing safety might curtail their freedom of movement and vice versa. Such an unintended consequences was observed when evaluating SSAGE in northeast Nigeria. SSAGE messaging that emphasized the important of keeping girls safe was sometimes internalized by caregivers and girls’ older adolescent brothers as a call to limit girls’ mobility, particularly at night (Koris et al., 2022).
Ensure pathways to scale and sustainability within the initial program design (Safe at Home)	• For Safe at Home, the largest cost category (52%) was directed towards Congolese protection staff that provided supportive supervision for community facilitators, led community mobilization, and provided case management services for survivors. Given the community-driven nature of the intervention, economies of scale may be difficult but could be achieved through greater intensity of services in targeted areas or through technologically supported supervision and ongoing trainings, which may be more feasible as the program is implemented repeatedly by facilitators It is also likely costs would vary greatly based on humanitarian setting as local wages heavily drive implementation costs.

## Program overview

### Safe at home

The Safe at Home program comprises community-driven discussion groups designed to address and prevent IPV and child maltreatment in conflict-affected communities.^15^ The initiative involves separate discussion groups for men and women on a weekly basis, along with monthly family discussion groups for couples and children. The program spans six to eight months, comprising 24 sessions for men, 18 sessions for women, and five family sessions. During weekly sessions, participants critically reflect on and engage in dialogues about gender, power dynamics, and privilege. They also receive education on the causes and consequences of violence against women (VAW) and VAC, while acquiring skills in stress management, psychosocial support, and positive parenting. Family sessions emphasize enhancing relationship quality, fostering shared decision-making among partners, and involving children in family decision-making. The program underwent a pilot phase with a pre–posttest in 2017–2018 and was tested through a pilot cluster-randomized controlled trial between 2019 and 2021. Results showed promise in reducing IPV and VAC, as well as improving the hypothesized mechanisms of change.^15^

## Sibling support for adolescent girls in emergencies

SSAGE is a whole-family, gender-transformative program designed to improve family functioning, disrupt intergenerational cycles of violence, and proactively mitigate future instances of violence against adolescent girls. The program engages four family members, including adolescent girls, their male siblings, and both male and female caregivers. For 12 weeks, participants attend weekly synchronized sessions with interactive curricula tailored to their respective age and gender categories. Sessions are guided by gender-transformative pedagogy and facilitated by a same-gender mentor, covering topics such as violence, gender norms, decision-making and power, and others. Participants are encouraged to discuss each week’s topics with their participating family members at home. In each new implementation setting, content adaptation is informed by a community-based participatory approach to ensure the program is culturally relevant, acceptable and appropriate. To date, the program has been evaluated, using mixed methodologies, with internally displaced populations in Northeast Nigeria, Syrian refugees in Jordan, and refugee and host populations in Niger. Preliminary evidence points to improved family functioning, healthier and safer family relationships, and improved well-being for adolescent girls.^16^^,^^17^

## Recommendation #1 recognize the diversity of families in humanitarian settings

Recognizing the diversity of families is a critical consideration when developing GTPP to address GBV. The upheaval caused by crises can disrupt family structures and norms; understanding these changes is important for identifying caregivers for children, including if functional caregivers have shifted due to the humanitarian crises, as well as improving program content to reflect diverse family units. Program targeting must recognize the parent-child structure might not be ubiquitous and that non-traditional caregivers can play a significant role in children’s lives.^18^

Non-traditional caregivers can include extended family members, older siblings, neighbors, or community members or leaders such as religious officials. In humanitarian settings, diversity extends not only to the nature of caregiving but also to the myriad challenges faced by different caregivers due to the disaster. For example, younger caregivers, including older siblings, may have also experienced recent exposure to violence that requires attention. In alignment with this approach, programming should consider targeting vulnerable groups of caregivers who may be particularly impacted by the crisis, when relevant to the program and when there is minimal risk of inadvertently creating stigma.

While gender-aware programming may recognize different caregiver groups, gender-transformative programming must actively consider the implications of various caregiving roles in humanitarian contexts and directly work with various caregiving groups. While caregiver groups may need to be stratified by role or generation in addition to gender across settings, GTPP in humanitarian contexts may also need to consider risk profiles for conflict exposure by parenting groups. For example, sibling-caregivers may also require parenting programmatic considerations that address implications of parental loss alongside the gendered parenting norms of the absent biological parent(s).

It is essential to acknowledge that there is not a one-size-fits-all approach in any context, as the intersectional risks and feasibility and acceptability issues will inevitably vary among family members and structures. This is particularly important in humanitarian contexts where fundamental familial and societal shifts may have impacted caregiving expectations and roles within the household and across the community. Caregivers participating in SSAGE expressed appreciation for the inclusion of adolescent males in the program, a group that would often be overlooked or irrelevant in development settings, noting that this gender transformative approach urged them to reflect on the different ways in which they parent boys versus girls; these parenting practices may not have been as readily examined if the program engaged adolescent girls only. Further, whole-family programs like SSAGE and Safe at Home can be instrumental in addressing the needs of the entire family to yield more holistic and sustainable results. Safe at Home’s ‘power ladders’ for gender and age, for example, highlight the differences in younger versus older children, creating age-appropriate content and discussions to cater to the unique needs of family members.^15^

## Recommendation #2: prioritize participatory approaches from the start

Practitioners and researchers in are increasingly calling for community-driven interventions that comprise formal and informal services and partnerships.^19^ Community-driven approaches are particularly relevant in humanitarian contexts where international organizations may not have an extended presence in a community. Communities’ needs, perspectives, and contexts are diverse and dynamic, particularly as norms shift during humanitarian crises, and any GTPP must be adapted to reflect the unique risks and protective factors affecting men, women, and children, while also acknowledging the potential for rapid contextual shifts throughout a humanitarian crisis.

Employing participatory approaches throughout the program cycle that are responsive to contextual shifts, from design to analysis and the dissemination of findings, is critical for maximizing program acceptability, effectiveness, and sustainability.^20^ Unlike in development settings, enabling participatory approaches requires unique considerations that reflect accessibility challenges and mobility realities of humanitarian contexts. At the same time, it is critical that adaptations are well documented and that fidelity to evidence-based core content is retained. Moreover, children’s input, especially girls’, can be invaluable in ensuring that the needs of children and families are met.

Following an initial implementation of SSAGE in Northeast Nigeria, for example, program implementers led a participatory contextualization process in two new settings (Jordan and Niger) prior to the program’s start. This process involved conducting focus group discussions with adolescent boys, male caregivers, and female caregivers as well as participatory activities with adolescent girls.^21^ Recognizing the acute risk of research fatigue in this humanitarian context, focus group discussions included were designed to be unique from standardized approaches. Gender-aware focus group discussions were used to elicit family members’ perceptions of brother-sister relationships, differences in boys’ and girls’ lives, and parenting styles with respect to children’s gender. Participatory activities with adolescent girls, which were designed with all levels of literacy in mind, enabled them to share the nature of their family relationships, safety concerns, and strengths. Findings from these activities were able to inform gender-transformative iterative program adaptations, to address these varied and emergent parenting styles in this humanitarian context, that were shared with the community for additional feedback and ongoing input.

An accountability mechanism should also be woven into program design and implementation to ensure programmatic adjustments are made based on the ongoing participation of the community. These mechanisms need to be responsive to the humanitarian context, through considering access despite contextual shifts (incl. mobility or displacement of participants). Program design should incorporate feedback loops that encourage community engagement and participation throughout the program’s lifecycle, promoting ownership and responsiveness, while also being receptive to how displacement and conflict may alter the composition of the community. For example, within Safe at Home, it was critical to separate men’s and women’s discussion groups as community insight and previous programming documented how men’s voices would dominate discussions and topics in shared groups between men and women. Therefore, Safe at Home provided separate spaces for men and women; thus, enabling women’s voices to be centered (gender-aware) and where a feedback loop was created by facilitators to ensure that topics of men’s groups remained accountable to the needs of women (gender-transformative).

## Recommendation #3: set realistic parameters and goals for the specific humanitarian context

Conducting gender analyses is important to amplify results, while also preventing the risk of inadvertently doing harm. In conflict and post-conflict settings, the inherent disruption in economic stability and access to essential goods and services can lead to a range of direct and indirect stressors. These stressors can exacerbate and perpetuate harmful norms,^3^ further magnifying the risks and impacts of GBV.^15^ In addition to the elevated risk of GBV,^4^ there may be in an increased risk of early or forced marriages for girls in some settings alongside other context-specific GBV risks.^22^^,^^23^

In areas where extreme insecurity is likely to obstruct curriculum rollout, careful consideration should be given to the implementation strategy. Exploring digitally flexible approaches, either in complement to face-to-face programming or as standalone programming, becomes increasingly significant in contexts of displacement or rapid changes; however, it is important to recognize when such approaches pose their own safety’s risks.^24^ Adaptations for acute emergency settings should be paired with theories of change that are realistic in what is achievable with limited participant engagement and prioritize the safety and wellbeing of women and children.

Rapid and volatile changes in humanitarian contexts necessitate thorough consideration of program feasibility, including its ability to affect gender transformative results, and its ethical dimensions, especially the safety of women and girls. Of particular concern is the time requirements for implementation of GTPP that grapple with deeply rooted behaviors and attitudes. Programs like Safe at Home (18–24 weeks), SSAGE (12 weeks) and others, must consider whether caregivers will be able to remain in the area for the duration of the intervention. Another crosscutting consideration is the sufficiency of ongoing and anticipated resource mobilization within the programming timeframe, including trained staff for implementation and the minimum standards to support survivors.^25^^,^^26^

These considerations can be integrated within a comprehensive risk assessment and service mapping, which is critical in humanitarian contests where the prevalence of GBV is often higher and resources more limited.^4^^,^^27^ The SSAGE mentor training toolkit, for instance, describes guidelines and minimum resource requirements related to psychological first aid, handling of disclosures, referrals to other specialized services, child safeguarding, and protection from sexual exploitation and abuse. If basic services - like sexual and reproductive health services, child protection, and GBV case management – or the capacity to provide them are unavailable, program implementation may need to be reconsidered. Without proper management of referral and follow-up, survivors’ needs may be overlooked, trust among participants may erode, and the overall effectiveness of the program may be compromised. Even after programming has started, it is possible that the need for services may be higher than anticipated and that additional staff may need to be hired to join session. For example, SSAGE needed to pause data collection in order to find a social worker that could join remotely to do psychosocial first aid because the disclosures and distress were much higher than anticipated. Finally, service mapping can both support referrals to the parenting programming for families where risk factors are identified by other services as well as be used to disseminate broad messaging that would reinforce learnings and/or reach those families who have not been recruited into the program. It is especially important in humanitarian settings that service mapping is ongoing, to reflect the potential for rapid shifts in availability of services.

Facilitators must have a deep understanding of the context and the different risks that arise from the extreme pressures of humanitarian settings. Facilitation teams must be proactive in tracking attendance and identifying trends, with preplanned approaches to make adjustments to maximize participation. It is also crucial to promptly identify contextual changes such as policy shifts or direct exposure to conflict that may necessitate changes in programming, including early program termination.

## Recommendation #4: ensure pathways to scale and sustainability within initial program design

Promoting gender-transformative thinking and addressing GBV is a complex endeavor that transcends the limitations of program cycles, particularly abbreviated humanitarian program cycles. Importantly, gender-transformative change does not happen in a vacuum or overnight. Recognizing what may be a departure for traditional humanitarian practice and current funding mechanisms, the sector needs to shift to foster sustainability beyond the program cycle, ensuring that the impact endures well into the future and considers the humanitarian-development nexus. Similar to development settings, where national parenting programs already exist, partners should adapt these programs rather than create separate programming.

A crucial component of sustained practice may involve engaging local women’s and youth organizations, both longstanding organizations and those that emerged after crisis onset. Collaborating with these organizations can involve building on local capacity and shifting away from a model where interventions are led by international non-governmental organizations. However, unlike in development settings, contingency considerations need to be made to prepare and respond to shifts in access and community composition that may impact these organizations. Shifting power to local voices can allow adaptations, implementation strategies, and sustainability discussions to be led by these groups from the start and result in productive feedback loops that are responsive to ongoing societal and community shifts. Some male caregivers participating in the Nigeria implementation of SSAGE were also members of embedded male community groups, in which they shared lessons learned during SSAGE. Caregivers reported that such discussions served to socialize the messages shared in SSAGE and elicited excitement among male community members to participate in future implementations. Prior to implementation, organizations should identify community groups and other modalities of knowledge dissemination. This is especially important in humanitarian settings where informal pathways of knowledge sharing may need to be leveraged in the absence of external programming.

It is also important to promote discussions with policy makers both in-country and within the humanitarian-development nexus to plan dissemination and advocacy strategies alongside sustainability planning. This step includes concretizing the program’s recommendations into policy after the program concludes, perhaps aligning VAW and VAC efforts when shared drivers are identified, to inform sustained and embedded gender transformation. Advocacy can help sustain the momentum and ensure that gender-transformative thinking becomes an integral part of the policy framework, amplifying the impact beyond the program’s initial reach and moving beyond gender awareness. Importantly, conducting robust evaluations of programs—as was done with SSAGE and Safe at Home—is vital for advocacy and demonstrating to implementing partners and donors the need for and feasibility of gender transformative approaches compared to gender insensitive, or even gender-aware, programming.

Finally, analyzing the major cost drivers within a program can offer insights into potential adjustments to delivery modalities that might reduce costs without compromising quality and effectiveness. While costing is relevant across contexts, it may be particularly important in humanitarian contexts where funding streams drastically reduce as a crises protracted. This data-driven approach can optimize resource allocation and streamline program implementation. Moreover, leveraging technology can provide cost-effective ways to deliver programming, reach more people, and reduce overall costs. It is important to identify suitable technological solutions, when relevant, and incorporate them strategically into program design.

## Important considerations and next steps

GTPP is emerging, but there is still meaningful room for growth in humanitarian settings, specifically. We propose four recommendations for GTPP in humanitarian context, with the specific interest to improve outcomes specific to IPV and VAC. It is our hope that these recommendations can be useful to programmers and policy makers in building effective, scaled, and evidenced-based parenting programming in humanitarian settings.

## Contributors

CP, IS, LS, and MM conceptualized the study. IS and MM conducted the review. MM wrote the initial commentary draft with CP, DR, IS, JD, KF, and LS providing input across drafts. All authors approved the final commentary.

## Declaration of interests

The authors do not report any conflicts of interest.
